# Δ^2^ machine learning for reaction property prediction†

**DOI:** 10.1039/d3sc02408c

**Published:** 2023-07-19

**Authors:** Qiyuan Zhao, Dylan M. Anstine, Olexandr Isayev, Brett M. Savoie

**Affiliations:** a Davidson School of Chemical Engineering, Purdue University West Lafayette IN 47906 USA bsavoie@purdue.edu; b Department of Chemistry, Carnegie Mellon University Pittsburgh PA 15213 USA olexandr@olexandrisayev.com

## Abstract

The emergence of Δ-learning models, whereby machine learning (ML) is used to predict a correction to a low-level energy calculation, provides a versatile route to accelerate high-level energy evaluations at a given geometry. However, Δ-learning models are inapplicable to reaction properties like heats of reaction and activation energies that require both a high-level geometry and energy evaluation. Here, a Δ^2^-learning model is introduced that can predict high-level activation energies based on low-level critical-point geometries. The Δ^2^ model uses an atom-wise featurization typical of contemporary ML interatomic potentials (MLIPs) and is trained on a dataset of ∼167 000 reactions, using the GFN2-xTB energy and critical-point geometry as a low-level input and the B3LYP-D3/TZVP energy calculated at the B3LYP-D3/TZVP critical point as a high-level target. The excellent performance of the Δ^2^ model on unseen reactions demonstrates the surprising ease with which the model implicitly learns the geometric deviations between the low-level and high-level geometries that condition the activation energy prediction. The transferability of the Δ^2^ model is validated on several external testing sets where it shows near chemical accuracy, illustrating the benefits of combining ML models with readily available physical-based information from semi-empirical quantum chemistry calculations. Fine-tuning of the Δ^2^ model on a small number of Gaussian-4 calculations produced a 35% accuracy improvement over DFT activation energy predictions while retaining xTB-level cost. The Δ^2^ model approach proves to be an efficient strategy for accelerating chemical reaction characterization with minimal sacrifice in prediction accuracy.

## Abstract 2

Newly developed Δ^2^-learning models enable state-of-the-art accuracy in predicting the properties of chemical reactions.

## Graphical abstract

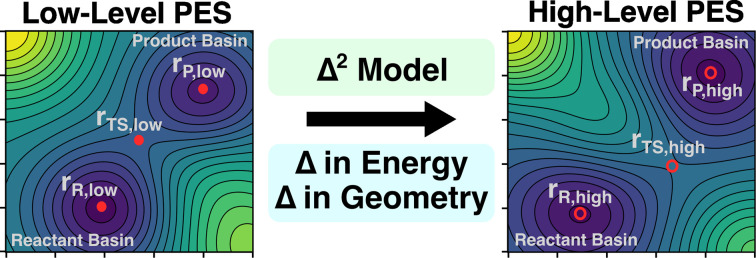

## 1 Introduction

Automated reaction network prediction can elucidate key reaction mechanisms, predict reaction outcomes, and guide catalyst design toward improving the yield of valuable products.^1–3^ Unlike traditional network exploration algorithms that are based on encoded reaction types, automated reaction prediction methods can discover unexpected reaction mechanisms and new reaction types with limited use of heuristic rules.^4,5^ The use of automated reaction prediction is widespread and has been successfully applied in research areas such as biomass conversion,^6^ combustion chemistry,^7–9^ and heterogeneous catalysis.^10–13^ However, the main bottleneck of large-scale automated reaction prediction methods is the cost of locating transition states (TSs) on the atomic potential energy surface (PES). Even though various algorithms have been developed to accelerate TS searches, including single-ended searches (*e.g.*, AFIR^14^ and SSWM^15^) and double-ended searches (*e.g.*, NEB^16^ and string methods^17–19^), energy and force evaluations must be performed at high levels of theory to obtain accurate activation energies and reaction energies.

To overcome large computing requirements, machine learning (ML) approaches have been adopted in recent years to accelerate reaction-rate predictions by reducing the dependency on DFT calculations. The methods can be classified into three directions. The first approach is to use ML interatomic potentials (MLIPs) rather than high-level quantum chemistry to explore the PES. A variety of neural network (NN) based force-fields have been developed to simulate equilibrium structures, including ANI,^20^ AIMNet,^21^ PaiNN,^22^ and Allegro,^23^ among others. More recently, reactive MLIPs have been developed to locate the TS and explore the minimum energy pathway (MEP).^6,24–28^ However, there is a scarcity of large generalized reaction databases, and as a result, reactive MLIPs are currently limited to specific chemistries and configurations. The second approach is to predict the TS geometries based on the geometries of reactant(s) and product(s), thereby circumventing the PES exploration for a TS. For example, Pattanaik *et al.* trained a graph neural network to predict TSs of isomerization reactions, which shows a 71% accuracy in reproducing the DFT-level optimized TS.^29^ Makoś *et al.* trained a generative adversarial network on the same dataset, but did not further validate that the resulting TS matched to the input reaction.^30^ However, DFT-level refinements of the predicted TSs are still required to obtain accurate activation energies with the current limitations of these models. The third approach is to directly predict the activation energy based on the reactant and product.^31^ Heinen *et al.* and Stuyver *et al.* trained a kernel ridge regression model^32^ and a graph neural network^33^ on a dataset of ∼4000 S_N_2 and E2 reactions, respectively, achieving a mean absolute error (MAE) of 2.5 and 2.6 kcal mol^−1^. A series of models based on chemprop,^34^ a directed message passing neural network (D-MPNN), were trained on a more diverse reaction database generated by Grambow *et al.*^35^ When tested on an independent test set, the best MAE reached ∼3.0 kcal mol^−1^.^36–38^ Ismail *et al.* trained an artificial neural network (ANN) on the same dataset and reached a modestly lower MAE of ∼2.8 kcal mol^−1^ on a withheld testing set.^39^ One concern for models that only use 2D information is that they are incapable of predicting more than one transition state for a given reactant/product pair, which potentially makes them more sensitive to conformational biases present in their training data. Presently, ML models based on this strategy have yet to achieve both chemical accuracy (<1 kcal mol^−1^) and transferability beyond narrow types of chemical species.

There are two factors that limit the predictive accuracy of ML models applied to reaction properties. One is the limited amount and/or accuracy of the available training data. The datasets used for training the aforementioned ML models contain only up to 12 000 reactions and did not use conformational sampling to identify the lowest energy TSs for each reaction. The second limitation is the amount of chemical information that can be derived from the input representation. Common molecular fingerprints (*e.g.*, Morgan fingerprints^40^) fail to capture information about atomic sequences, while representations based on two-dimensional molecular graphs do not distinguish between conformations. The emergence of reaction databases with larger chemical coverage and conformational sampling, such as the Reaction Graph Depth 1 (RGD1) database,^41^ mitigates the first limitation. Adopting a 3D molecular representation would eliminate the second.

**Fig. 1:**
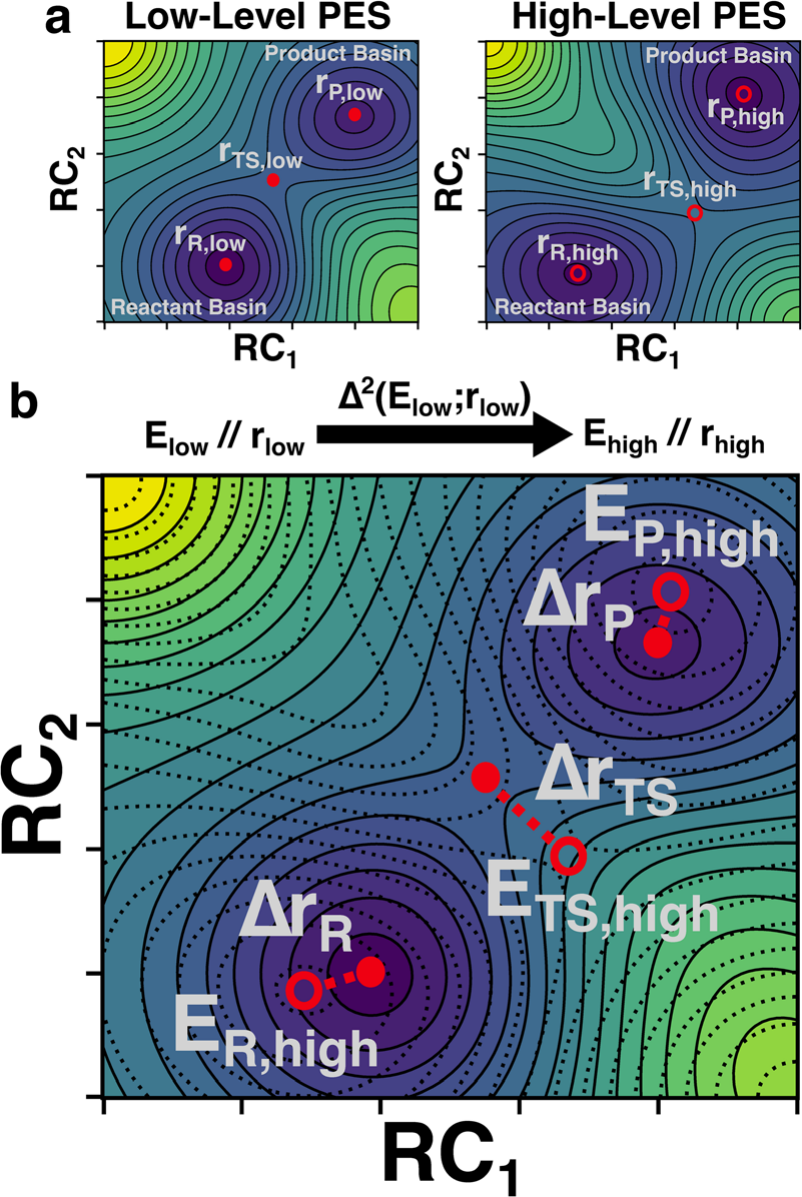
Overview of the Δ^2^-learning task. (a) Comparison of critical points on low-level (left) and high-level (right) potential energy surfaces. (b) The task of the Δ^2^ model is to predict the high-level energies at the high-level critical points (*E*_high_//*r*_high_) using only the low-level energies calculated at the low-level critical points (*E*_low_//*r*_low_). The differences in geometry are illustrated and the high-level PES, which is unknown at the time of prediction, is shown as a dotted overlay.

In this study, we develop a ML model that uses optimized geometries and energies calculated at a low-level of theory (*e.g.*, semi-empirical quantum chemistry) to predict the energies of structures optimized at an analogous critical point at a high-level of theory (*e.g.* density functional theory) (Fig. 1). The critical point on the potential energy surface (PES) is either an equilibrium structure (ES) or a TS and may show large geometric deviations between the low and high levels of theory (Fig. 1a). Despite these deviations, the model is tasked with predicting the high-level energy at the high-level critical-point based only on the low-level inputs (Fig. 1b). We refer to our trained predictor as a Δ^2^ model, where the Δ in geometry is learned implicitly and the Δ in property (*i.e.*, energy in this case) is learned explicitly. In contrast, Δ models typically only learn a difference in property (*e.g.*, an energy difference at a given geometry) without simultaneously learning a difference in geometry.^42,43^ Our findings show that the Δ^2^ model, trained on ∼167 000 reactions, achieves accurate predictions of the activation energy in both internal and external test sets. Fine-tuning the model on 2000 reactions computed at the Gaussian-4 (G4) level of theory further reduced the prediction errors by ∼1.5 kcal mol^−1^ compared with direct DFT calculations. In addition, the benefits of the Δ^2^ model in distinguishing different reaction conformations and accurately predicting the lowest activation energy are illustrated by a subset of test reactions.

## 2 Methodology

### 2.1 Database

The Δ^2^ model was trained using the RGD1 dataset, which contains ∼210 000 distinct reactions with up to ten heavy (carbon, nitrogen, and oxygen) atoms.^41^ The RGD1 database covers a diverse reaction space because it is generated by a graph-based exploration without the use of encoded reaction types. For a detailed description of the RGD1 database, we direct readers to our previous publication.^41^ Here, we briefly recapitulate the central steps of database generation that are relevant to the data processing in this work. RGD1 generation started with a graphical enumeration of 700 000 “breaking two bonds, forming two bonds (b2f2)” model reactions from reactants sampled from PubChem. A reaction conformational sampling strategy^44^ was applied to generate up to three conformations for each enumerated reaction, followed by a doubled-ended TS search, Berny optimization, and intrinsic reaction coordinate (IRC) validation at the GFN2-xTB^45^ level of theory. The GFN2-xTB optimized TSs were further refined using Gaussian16 quantum chemistry engine^46^ at the B3LYP-D3/TZVP^47–50^ level of theory. After DFT-level optimization, there is a chance that the TS may no longer correspond to the same reaction as the xTB-level TS (*i.e.*, the TS corresponds to an “unintended” reaction, because it does not connect the same reactant and product identified by the GFN2-xTB level IRC calculation). The DFT-level TSs were classified as intended or unintended using an XGBoost model that exhibits a testing set accuracy of ∼95%.

Several data processing steps were applied to prepare the RGD1 data for training the Δ^2^ model. First, TS data points were only considered duplicates if both the xTB-level and DFT-level TSs were identical (as opposed to only if the DFT-level TS was identical). This was done because the Δ^2^ model uses the GFN2-xTB geometry as an input and so retaining distinct xTB-level TSs that converge to same DFT level TS is useful for learning the implicit deviations between these geometries. Second, only intended TSs that correspond to the same reaction at both the DFT-level and xTB-level were used to train the Δ^2^ model. Unintended TSs cannot be used for training a Δ^2^ model, because the xTB-level TS does not correspond to the same reaction as the DFT-level TS in such cases and so a reference ground truth label is unavailable. Third, an additional effort was made to remove unintended TSs from the training data that might have been misclassified as intended by the XGBoost model. A 5-fold cross-validation (CV) was performed on all TSs to identify outliers. After training and testing the Δ^2^ model (*vide infra*) on each fold, each TS appeared once as a test data point and the corresponding deviation between the predicted energy and the reference value was defined as the deviation for each TS. IRC calculations were performed on 7242 TSs with a deviation greater than 10 kcal mol^−1^ (an empirically determined criterion). The IRC calculations identified 4057 of these TSs to be “unintended” and they were excluded from the dataset. In total 185 893 distinct reactions (only forward reactions are counted in this number, forward and reverse would be 2×) passed these filters and were used to train and test the Δ^2^ model. The entire dataset is provided as ESI.†

Among the 185 893 reactions, 122 964 are compositionally unique (*i.e.*, they have a unique combination of reactant(s) and product(s)), while the other 62 929 represent different TS conformations for reactions contained within the 122 964. A 90-10 training and testing split was applied to the 122 964 unique reactions. When creating these splits, reactions with different conformations were partitioned to the training or testing set as a group to ensure that the testing set was composed of unseen reaction compositions and reaction conformations.

The Δ^2^ model is trained on the geometries and energies associated with critical points, not on the reactions or activation energies directly. Thus each reaction in the testing and training set is associated with multiple geometries: the TS and the ESs of the individual reactants and products. To ensure that each chemical structure was exclusively assigned to either the training or testing set, only ESs exclusively appearing in the training reactions were included in the training set, while all other ESs, including those shared between the training and testing reactions, were assigned to the testing set. The shared ESs are a result of certain reactants or products being involved in multiple reactions, which also accounts for the similar number of unique ESs (122 856) and unique reactions (122 964). The resulting testing set thus exclusively contains unseen ESs and TSs corresponding to unseen reactant–product pairs. In total 273 687 chemical structures (167 269 TSs and 106 418 ESs) and 35 062 chemical structures (18 624 TSs and 16 438 ESs) were included in the training and testing sets, respectively.

All model performance results reported in the figures are for testing set predictions. For the activation energy predictions, the testing set contains activation energies of both forward and reverse directions. For enthalpy of reaction predictions, only enthalpies of reaction of the forward reactions are reported (the reverse are redundant).

### 2.2 Structural similarity

The Δ^2^ model is designed to predict properties computed at geometries optimized at a high-level model chemistry based on analogous properties and geometries optimized with a low-level model chemistry. A potentially important factor that affects the prediction accuracy is the magnitude of geometric deviation between the low and high levels of theory. To quantify the differences in molecular geometries, mass-weighted root-mean-squared-displacements (RMSDs) between the aligned geometries at each level of theory were computed (Fig. S1a†). RMSD is a first-level analysis that is sensitive to the entire conformation of each structure, however, for chemical reactions, structural changes in the reactive portions of each TS are oftentimes the defining features. To better describe the structural similarity between the TSs calculated at each level of theory, the maximum reactive bond length change (MBLC) between the low-level and high-level TSs was also calculated. For the example reaction shown in Fig. S4,† two bonds between atoms A and B and atoms C and D break and two bonds between atoms A and C and atoms B and D form; the MBLC is 0.58 Å in this case and corresponds to the CD-bond, which shows the largest deviation between the two levels of theory. To avoid including data points with large geometric inconsistencies that suggest different reaction chemistry being described by GFN2-xTB and DFT, a threshold of ≤1 Å was applied for both RMSD and MBLC (*i.e.*, violating either criteria led to exclusion), resulting in the exclusion of 1.1% (3351 out of 308 749) of the datapoints in the RGD1 database. A similar threshold was also applied in a previous study.^51^ The distributions of RMSD and MBLC are provided in Fig. S1.† After RMSD and MBLC threshold screening, 270 723 (164 323 TSs and 106 400 ESs) and 34 675 (18 238 TSs and 16 437 ESs) data points were retained to form the final training and testing sets, respectively.

### 2.3 Model architecture

The Δ^2^ model applied in this work is a neural network (NN) potential constructed using a variant of the AIMNet2-NSE architecture.^52^ The salient features and differences relevant for the current work are described below. AIMNet2-NSE was developed using a message passing approach,^52^ where the model learns an abstract and flexible chemical representation that is iteratively updated based on the representations of neighboring atoms. Briefly, our AIMNet Δ^2^ model first converts the local atom-centered geometries into atomic environment vectors (AEVs). These AEVs take the form of atom-centered symmetry functions that describe the local chemical environment of an atom using a number of gaussian probes with a set of width and peak position parameters. Details of the AEV functional forms can be found in previous works.^20,21^ The AEVs calculated in this work are truncated at 7.0 Å and consist of 24 atom-centered symmetry functions. Atomic numbers are embedded into a 16-dimensional learnable space that serves as the abstract hidden state representation for message passing, which we will refer to as the atomic feature vector (AFV). AFVs are updated over two message-passing iterations prior to energy predictions. This number of message-passing steps was selected based on preliminary tests that showed sufficient prediction accuracy and revealed that additional iterations did not yield noticeable gains in performance. As a high-level overview, a single message-passing step consists of constructing so-called embedded AEVs that are then passed through a through a multilayer perceptron (MLP) to obtain an updated AFV. Specifically, the Δ^2^ model combines geometric (symmetry functions) and nuclear (atomic embeddings) information into embedded AEVs through a summation of outer products between the AEV components and AFVs of neighboring atoms. A set of embedded radial and vector AEVs are concatenated, flattened, and passed through a MLP to predict AFV updates.

As a modification to the original AIMNet-NSE implementation, atom-centered point charges are initially predicted with a separate message-passing neural network that also uses embedded AEVs as the input. This design decision was informed by our model testing, where it was found that a separate message-passing neural network could be quickly trained for robust initial charge estimates. These atom-centered point charges are concatenated alongside embedded AEVs during the Δ^2^ model forward pass, and they are updated *via* neural charge equilibration^52^ during every message-passing iteration. Each message-passing iteration has a dedicated MLP to encourage model flexibility. The MLP used in the final message pass creates a single vector, of size 256, for each atom that is passed through an additional MLP for predicting the atomic contributions to the total energy.

**Fig. 2:**
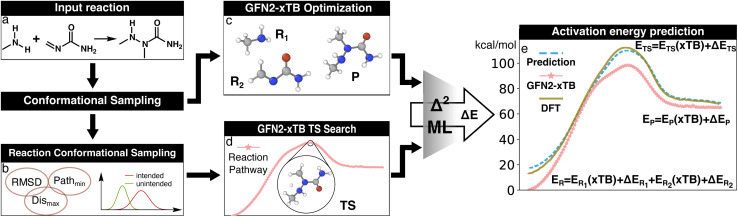
Illustration of the Δ^2^ Machine learning model architecture. For an input reaction (a), two workflows are applied to search for transition states (TS) and equilibrium structures (ES). To obtain the ESs, the separated reactant(s) and product(s) are subjected to conformational sampling and geometric optimization at the low level of theory (*i.e.*, GFN2-xTB in this study, c). The TSs are located by a reaction conformational sampling step that jointly optimizes reactant and product conformers for success in a double-ended search (b, see ref. 44 for more details), followed by double-ended TS localization at the low level of theory (d). The resulting geometries and energies are fed into the Δ^2^ model, which is built on an atom-wise featurized message-passing architecture. The output thermochemistry properties, like energies and enthalpies, are used to calculate the high-level activation energy and the enthalpy of reaction corresponding to the high-level geometries (e).

The Δ^2^ model was trained using the L2 loss of the total energy of the target species calculated at the low-level critical point (*i.e.*, either an ES or TS), where the ground truth values are the high-level of theory energies calculated at the high-level critical point (Fig. 2). Despite being common MLIP training practice, atomic force components were not used in the Δ^2^ objective function because the aim of the model is to infer the energy differences at PES critical points. Model training was carried out with a batch size of 100, the rectified Adam optimizer,^53^ and a cosine annealing scheduler^54^ that smoothly reduced the learning rate from 10^−4^ to 10^−6^ over 2000 epochs. To improve the quality of predictions, an ensemble of eight independent Δ^2^ models were trained with the same training dataset but different parameter initializations, and the inferred energies are reported as the average over the ensemble.

### 2.4 External test sets

Two external test sets were introduced to evaluate model transferability.^55^ The first contains three unimolecular decomposition networks of γ-ketohydroperoxide (KHP), propylene carbonate (PC), and methyl butanoate (MB). In total, 324 reactions containing up to seven heavy atoms (carbon, nitrogen, and oxygen) were collected from these networks after excluding reactions appearing in the RGD1 database. This external test consists of reactants of size and chemistry (CHON) similar to RGD1. The second external set contains 118 reactions possible in the first step of l-glucose pyrolysis. l-glucose (C_6_H_12_O_6_) contains 12 heavy atoms, which is beyond the molecular size of any species in the RGD1 database. In addition, the high reactivity introduced by multiple hydroxyl groups poses a unique challenge for predicting activation energies because such features are modestly represented in the training data.

### 2.5 Transfer learning for predicting G4 energies

The RGD1 database was constructed at the B3LYP-D3/TZVP level of theory. To explore the feasibility of using transfer learning to achieve higher accuracy for the Δ^2^ model, 2000 reactions were randomly selected to perform Gaussian-4 (G4) calculations (without geometric optimization) on the reactant, product, and TS geometries.^56^ In total 2000 TSs and 3142 corresponding unique reactants and products (ESs) were computed by the G4 method. Although G4 is one of the most accurate single reference methods, G4 calculations have a dissuadingly large computational cost. Thus, ∼1% of the training dataset was selected to perform these calculations and fine-tune the pre-trained xTB-DFT Δ^2^ model to a higher level of accuracy with transfer learning.

## 3 Results and discussion

**Fig. 3:**
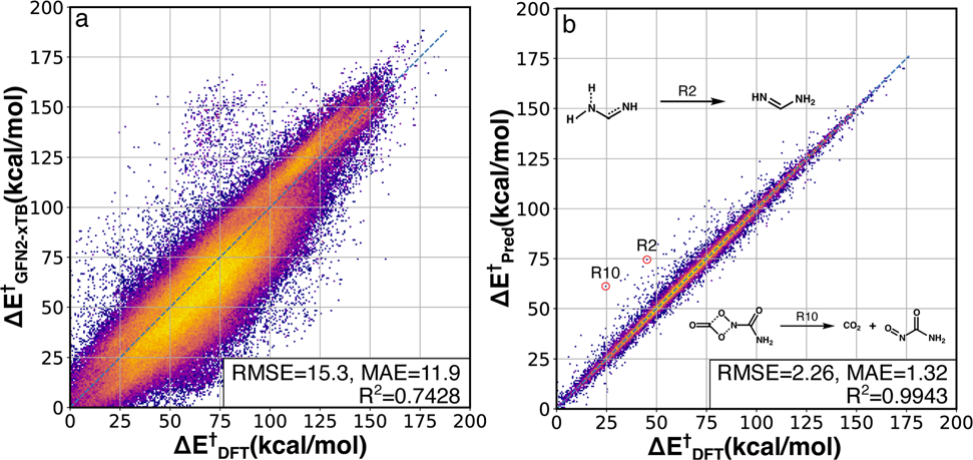
DFT-baseline and Δ^2^ model performance on the RGD1 testing set. Correlation between activation energies computed using GFN2-xTB (a) and the Δ^2^ model (b) with the ground-truth DFT values (*x* axes). The datapoints are colored based on bivariate kernel density estimations (yellow: high, purple: low). For each plot, units for both axes, RMSE, and mean absolute error (MAE) values are in kcal mol^−1^. 2 out of 14 outliers with prediction error larger than 20 kcal mol^−1^ are shown in the inset, while other outliers are provided in Fig. S3.†

The activation energy (Δ*E*^†^) deviations between GFN2-xTB and B3LYP-D3/TZVP were computed for the whole RGD1 database, which serves as a baseline comparison of the Δ^2^ model (Fig. 3a). GFN2-xTB typically underestimates the activation energies of lower barrier reactions (Δ*E*^†^ < 120 kcal mol^−1^) and overestimates the activation energies of higher barrier reactions (Δ*E*^†^ > 120 kcal mol^−1^). This inconsistency leads to an overall MAE of 11.9 kcal mol^−1^ and the difficulty of estimating the deviation through a straightforward linear regression. The Δ^2^ model improves the Δ*E*^†^ prediction accuracy by a factor of ∼7−9 (Fig. 3b), where the root-mean-square error (RMSE) and mean absolute error (MAE) were reduced from 15.3 and 11.9 kcal mol^−1^ to 2.26 and 1.32 kcal mol^−1^, respectively. Moreover, the Δ^2^ model implicitly learns to correct the range specific over/underestimations of the direct GFN2-xTB prediction, which can be seen in the increase of the *R*^2^ score from 0.74 to 0.99. Considering the wide span of the reaction space and the large range of target Δ*E*^†^ values (0–188 kcal mol^−1^), the MAE of 1.3 kcal mol^−1^ is an encouraging result, indicating that the Δ^2^ model can outperform alternative models using only information from reactants and products.^32,36,39^ Out of 36 358 test reactions (including both forward and backward pathways), only 249 reactions had an absolute error over 10 kcal mol^−1^, in other words, 99.3% of predicted Δ*E*^†^ were within a 10 kcal mol^−1^ deviation range (96.2% and 81.0% are within 5 and 2 kcal mol^−1^, respectively). A detailed analysis of 28 reactions (14 TSs) with absolute errors exceeding 20 kcal mol^−1^ is provided in Fig. S3.† In addition to the activation energy prediction, the Δ^2^ model was also applied to predict the enthalpy of reaction resulting in an even better MAE of 0.58 kcal mol^−1^ (Fig. S2†).

**Fig. 4:**
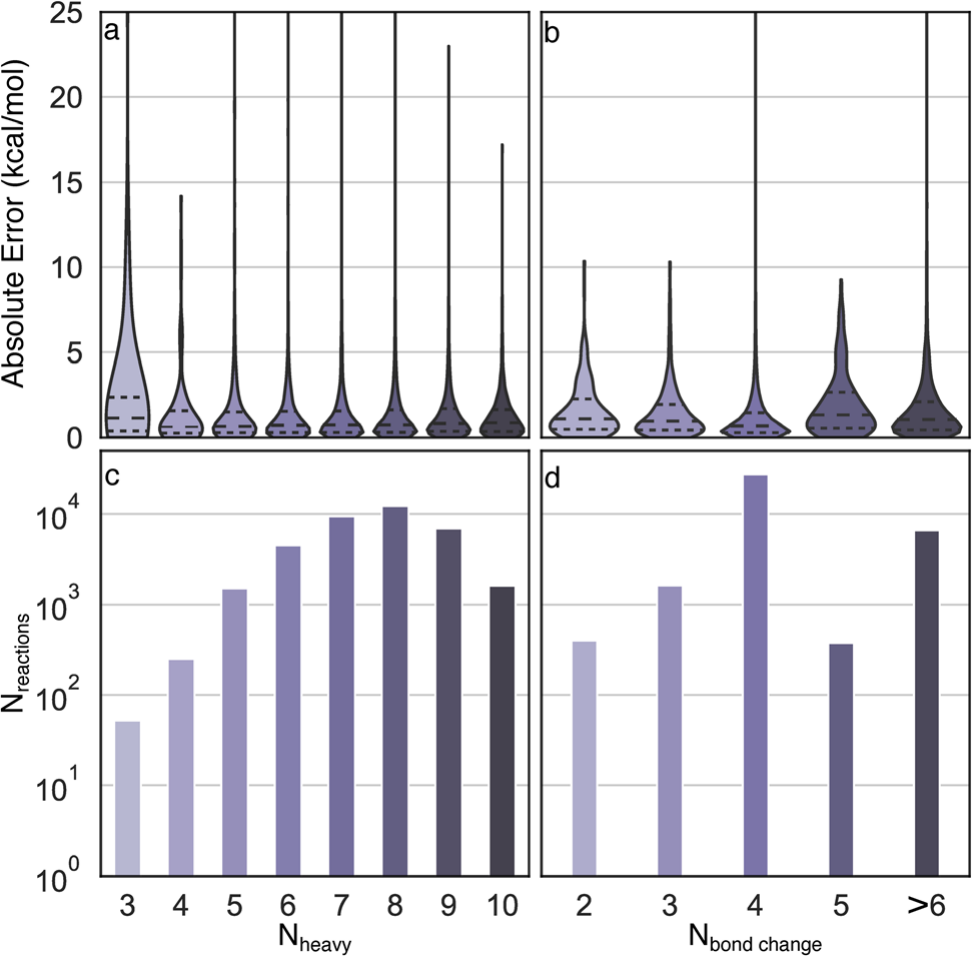
Analysis of error distributions as a function of chemical composition. Distributions of absolute errors by (a) number of heavy atoms and (b) number of bond changes involved in each reaction. The distributions are scaled to have equal width. The corresponding number of reactions with different number of heavy atoms and bond changes are shown in panel (c) and (d).

Uniform error distributions were observed across different sizes and types of reactions (Fig. 4), which is a common challenge for recently reported models.^36^ The reactions with three heavy atoms have a relatively large MAE of 3.2 kcal mol^−1^, which is mainly due to the lack of training data and one outlier in the test set (R2 in Fig. S3†). However, it is worth highlighting that the lack of accuracy of these systems is moot because directly performing DFT calculations of small species is a trivial task with modern computational resources. Consistently low MAEs (<1.4 kcal mol^−1^) can be obtained for reactions with four to ten heavy atoms, indicating the Δ^2^ model is insensitive to size across the diversity of reactions examined. For reactions with different numbers of bond changes, the MAEs of all types of reactions are below 2 kcal mol^−1^, indicating the Δ^2^ model exhibits transferability to numerous reaction mechanisms, despite training data scarcity for certain types. For example, the reactions with five bond changes and greater than six bond changes have MAEs of 1.94 kcal mol^−1^ and 1.66 kcal mol^−1^, respectively, which demonstrates that the Δ^2^ model can be applied to a wide reaction space.

**Fig. 5:**
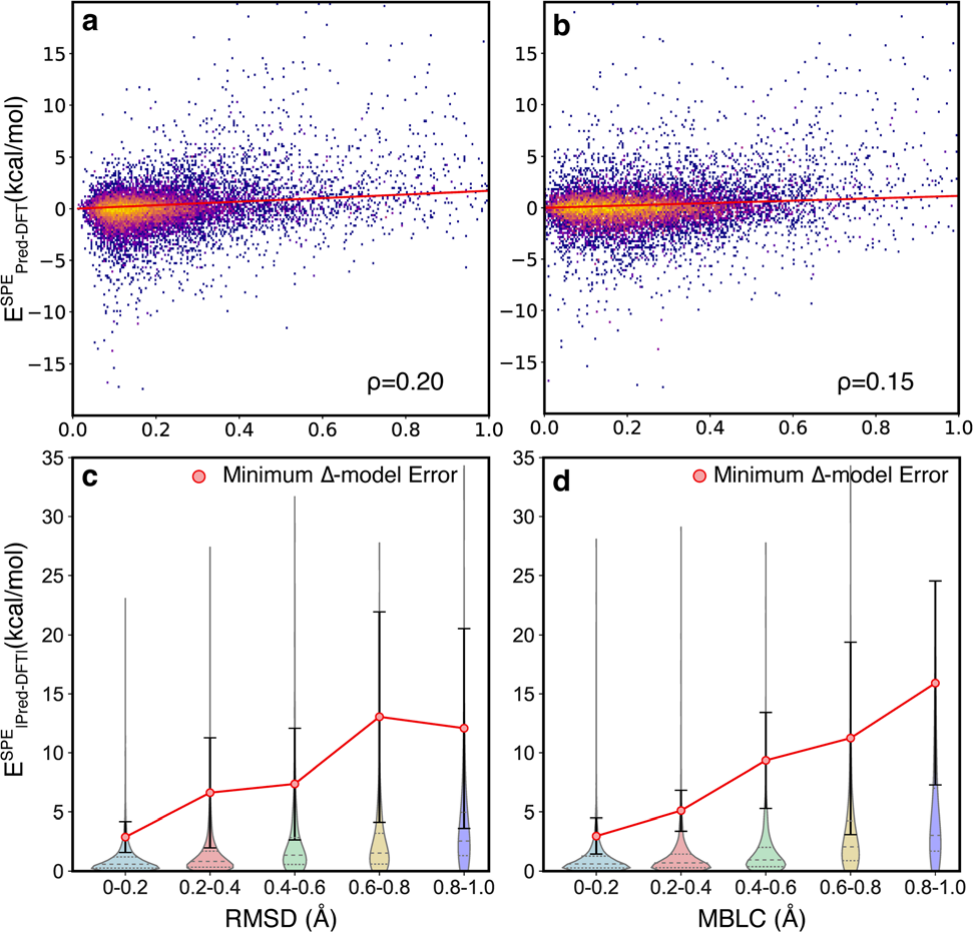
The effect of geometric deviations on the energy predictions. (a and b) The correlation between the signed error predicted by the Δ^2^ model and the RMSD and MBLC, respectively. The datapoints are colored based on bivariate kernel density estimations (yellow: high, purple: low). Least-squares linear regressions, with intersections anchored at zero, are presented as red lines. (c and d) The error distributions of the Δ^2^ model, binned by geometric deviations of RMSD and MBLC, respectively. For comparison, the mean and standard deviation of errors associated with using the DFT//xTB energies to approximate the DFT//DFT energies is shown as the red markers and whiskers, respectively.

The motivating hypothesis behind the development of the Δ^2^ model is that the geometric deviations between the low-level and high-level models can be learned implicitly while the energy deviations can be learned explicitly. This would be falsified by the observation of model errors that were strongly correlated with large geometric deviations between the low-level and high-level geometries, because this would indicate that the model is acting as a Δ model with predictions that are conditioned on high congruence between the geometries. To examine the effect of geometric deviations on the prediction errors, two measures (RMSD and MBLC) of geometric deviations between GFN2-xTB optimized and B3LYP-D3 optimized geometries are compared jointly with the signed error of the energy predicted by the Δ^2^ model at ESs and TSs (Fig. 5). For both measures, the majority of the data points are around the center line, *i.e.*, the mean signed error of zero, and thus, there is no clear trend between prediction accuracy and structural differences. The Spearman's rank correlation coefficients (*ρ*) between the two measures of geometric deviations and the absolute error are similarly low, with *ρ* = 0.2 for RMSD and *ρ* = 0.15 for MBLC, and a least-squares linear fit shows a very weak linear trend.

To provide a quantitative measure of the size of the correlation between structural deviation and accuracy, the errors from the Δ^2^ model were compared with the errors associated with using the B3LYP-D3/TZVP//GFN2-xTB energies to estimate the B3LYP-D3/TZVP//B3LYP-D3/TZVP energies (Fig. 5c and d). This latter measure is the lower limit of the errors for a Δ model trained to predict energies at a given geometry rather than at different fixed-points. The comparison was calculated for two hundred TSs that were sampled to span the structural deviations shown in the figure (See ESI Section 5† for additional details). The differences in this comparison are qualitative, with the Δ^2^ model showing errors 3 − 5*x* lower that the theoretically perfect Δ model across the various distributions. These observations are consistent with the hypothesis that the size of the geometric deviation should not be a strong indicator of prediction error for the Δ^2^ model because the geometry differences between the low-level and high-level are implicitly learned during training.

**Fig. 6:**
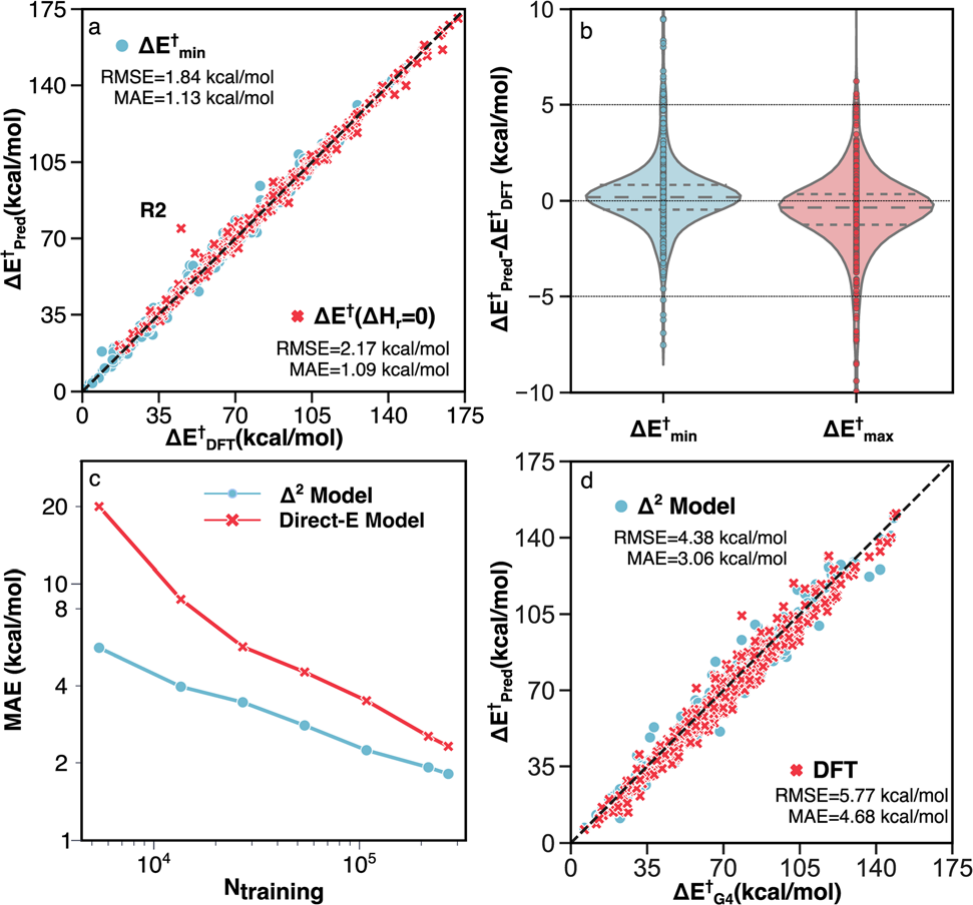
Performance of the Δ^2^ model on different subsets of testing data. (a) Parity plot of model predictions *vs.* DFT (B3LYP-D3/TZVP) data for reactions with identical reactants and products (red) and reactions with multiple TS conformations (blue). The outlier R2 is discussed in detail in Section S3.† (b) Error distributions for predicting the minimum (blue) and maximum (red) activation energies across multiple TS conformations. (c) MAE *vs.* the number of training data points for the direct-E model (red) and Δ^2^ model (blue). (d) Parity plot of activation energies calculated by G4 *vs.* DFT (red) and the Δ^2^ model trained with transfer learning (blue).

Two unique features of the RGD1 database are the inclusion of reactions that have chemically equivalent reactants and products but that still undergo bond rearrangements (denoted as Δ*H*_r_ = 0 reactions), and the inclusion of reactions with multiple transition state conformations connecting the same reactant and product. These two subsets of reactions challenge models based on molecular fingerprinting and general two-dimensional (*i.e.*, graph) molecular representations. The former cannot retain atomic mapping and will therefore return a “null” reaction, while the latter cannot describe conformational effects and is restricted to providing a single value prediction (*i.e.*, the reactant and product graphs have a one-to-many non-functional relationship to the different TS conformers). The Δ^2^ model can naturally handle these two types of reactions because its predictions are based on the 3D structure. Benchmark accuracy on these corresponding reactions of the test set are shown in Fig. 6a and b. The MAE and RMSE of 603 Δ*H*_r_ = 0 reactions are 1.09 and 2.17 kcal mol^−1^, respectively, while the target activation energy ranged from 15 to 170 kcal mol^−1^ (Fig. 6a).

For the 660 reactions with multiple TS conformations and a range of activation energies greater than 5 kcal mol^−1^, the Δ^2^ model accurately predicts both the lowest (Δ*E*^†^_min_) and highest (Δ*E*^†^_max_) activation energies (Fig. 6b) among the TS conformers. The MAE and RMSE of Δ*E*^†^_min_, which plays the most important role in modeling the kinetics of a reactive system with transition state theory (TST), are 1.13 and 1.84 kcal mol^−1^, respectively (Fig. 6a). The Δ*E*^†^_max_ is also accurately predicted with MAE and RMSE of 1.40 and 2.30 kcal mol^−1^, respectively. As a result, the Δ^2^ model is able to reproduce the range of activation energies (max–min) among different reaction conformations, which represents the effect of conformational sampling, with an MAE and RMSE of 1.83 and 2.79 kcal mol^−1^, respectively.

To assess the impact of the training dataset size on the model performance, the MAEs of the Δ^2^ model were calculated for training a single model from scratch using different amounts of training data and fixed hyperparameters (Fig. 6c). For comparison, a model was trained to directly predict the DFT-level fixed-point energy using the low-level fixed-point geometry, rather than the difference between low and high-level fixed points energies (“Direct-E model”, Fig. 6c). The difference in slopes is caused by the difference in complexity between learning the DFT energy directly (red) and learning the difference in fixed-point energy (blue). This creates the largest accuracy difference in the data-scarce regime, whereas with sufficient data, both models should asymptotically approach the same accuracy (*i.e.*, the accuracy limited only by the irreducible error of the dataset). Neither model shows evidence of accuracy saturation, suggesting further opportunities for dataset curation.

Since the accuracy of a ML model is bound by the accuracy of the training data, *i.e.*, the accuracy of B3LYP-D3/TZVP in our case, including higher accuracy reference data is one strategy to improve the inference of the Δ^2^ model. A transfer learning approach was applied with G4 calculations on 2000 TSs and 3142 equilibrium structures, which corresponds to ∼1% of the entire DFT dataset. A comparison between the G4 ground-truth data, B3LYP-D3/TZVP DFT, and the transfer learning Δ^2^ predictions are shown in Fig. 6d. We observe that including a small amount of G4 data (5142 data points) enables the ensemble of five Δ^2^ models to achieve a MAE that is 1.62 kcal mol^−1^ lower than DFT predictions on the G4 test data (3.06 compared 4.68 kcal mol^−1^, respectively, Fig. 6d). It is important to highlight that the transfer learning Δ^2^ model operates on GFN2-xTB geometries, meaning that the accuracy improvement of 35% compared to the DFT calculation only requires xTB-level cost (as shown in section S4, the runtime of the Δ^2^ model is negligible compared with xTB). The data efficiency of the transfer learning approach illustrates the high flexibility of the Δ^2^ model in that only a small amount of data is required to alter the target level of theory.

**Fig. 7:**
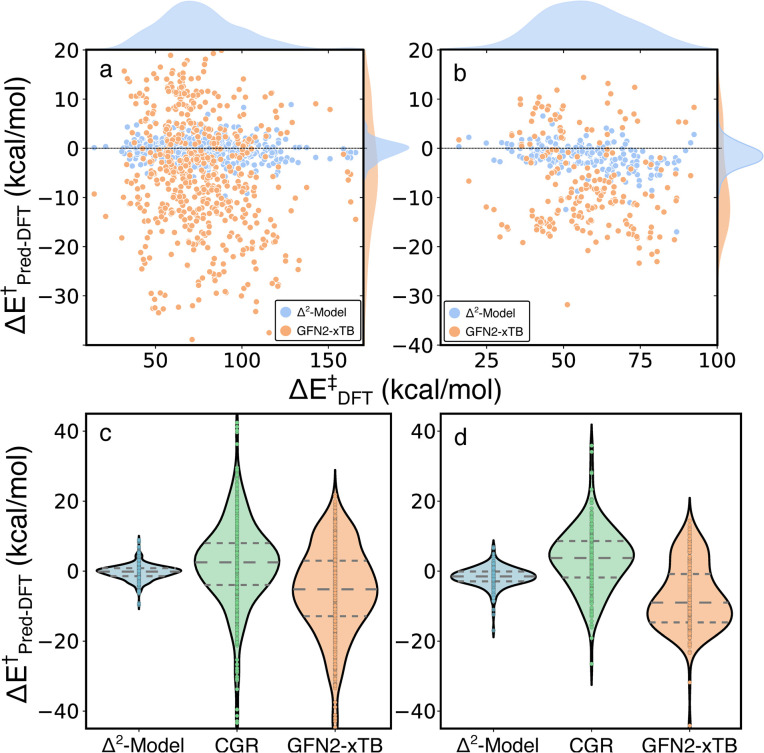
Performance of the Δ^2^ model on external testing sets. Error distributions of GFN2-xTB (yellow) and the Δ^2^ model (blue) on (a) unimolecular decomposition networks and (b) glucose pyrolysis reactions. Comparisons of the Δ^2^ model (blue), CGR model (green) and GFN2-xTB (yellow) on (c) unimolecular decomposition networks and (d) glucose pyrolysis reactions.

The Δ^2^ model was also applied to two external test sets, unimolecular decomposition networks (EXT1) and glucose pyrolysis reactions (EXT2), to evaluate model transferability (Fig. 7 and S4†). The mean signed error (MSE) of activation energies computed by GFN2-xTB with respect to those computed by B3LYP-D3/TZVP are −5.69, −7.57 and −5.71 kcal mol^−1^, in EXT1, EXT2, and the RGD1 database, respectively (the systematic bias can be clearly observed in Fig. 3a). The Δ^2^ model reduces the systematic MSE bias to −0.02 kcal mol^−1^ for EXT1 and reduces the MSE to −1.32 kcal mol^−1^ for EXT2, which is consistent with the corresponding deviation from the baseline theory (*i.e.* MSEs of −5.69, −7.57 kcal mol^−1^ in EXT1 and EXT2, respectively). The MAE of 1.52 kcal mol^−1^ in EXT1 indicates the transferability of the Δ^2^ model across the reaction space within ten heavy atoms (C, H, N, O), and the feasibility of using the model as a drop-in replacement for DFT in many reaction characterization tasks. A larger MAE of 2.33 kcal mol^−1^ is observed for EXT2, which we attribute to the systematic bias in EXT2 (*i.e.*, MSE of −1.32 kcal mol^−1^) and suggests that the transferability of the Δ^2^ model is limited by the baseline theory. A recently developed activation energy prediction model based on a representation of condensed graph of reaction (denoted as CGR, composed of an ensemble of five models) was applied to these two test sets.^37^ The CGR model combines a popular cheminformatics reaction representation, namely condensed graph of reaction, with a graph convolutional neural network architecture and reaches an activation energy prediction accuracy of ∼4 kcal mol^−1^ on a withheld testing set. The reaction dataset used to train the CGR model contains no more than seven heavy atoms,^35^ which is the same as EXT1 and is much smaller than EXT2. Although comparing models trained at different levels of theory is not ideal, the magnitude of the MAEs and uncertainties (9.38/2.94 and 7.51/2.32 kcal mol^−1^ for EXT1 and EXT2, respectively) are larger than can be explained by functional choice alone and indicates a lower transferability of the CGR model, which was solely trained on the 2D representations of reactants and products.

## 4 Conclusions and outlook

The maturation of machine learning methods in predicting reaction properties has created new opportunities in automated reaction prediction. Nevertheless, the relatively low accuracy and the lack of generality and transferability of existing models remain prohibitive for most applications. In this study, we have shown how these limitations can be side-stepped using the Δ^2^ model, which comes at the cost of GFN2-xTB but is able to provide beyond-DFT level accuracy. The performance of this model was investigated in four scenarios. First, the Δ^2^ model achieves a MAE of 1.3 kcal mol^−1^ on the withheld testing set, which is the most accurate prediction of activation energy by an ML model for general organic (C, H, O, N) chemical reactions to date. Second, the Δ^2^ model accurately predicts the activation energies of “null” reactions and reactions with multiple TS conformations. In particular, the ability to distinguish the activation energies of different reaction conformations and accurately predict the lowest activation energy is essential in reaction prediction tasks. Third, the generality of the Δ^2^ model was tested on two external test sets, one containing three unimolecular decomposition networks and the other involving the first step pyrolysis reactions of l-glucose. For reactions distributed in the same reaction space as the training dataset, the Δ^2^ model achieves similar performance, while for reactions outside the training data, the prediction error is slightly increased but still reliable. Finally, the transferability of the Δ^2^ model was demonstrated by altering the high-level target theory from DFT to G4. By fine-tuning the model with G4 values corresponding to only 1% of the training data, the model outperforms the prediction accuracy of DFT calculations on the same reactions.

There are still several avenues for improving the current approach. First, activation energy predictions that approach the chemical accuracy are only available for reactions containing up to ten C, N, and O atoms. To extend the accurate performance to a more diverse reaction space, either a larger, more complex reaction database or an ad-hoc transfer learning approach on additional specific datasets are needed. Second, the current model is only trained on neutral closed-shell species. There are no fundamental obstacles to extending the approach to ionic and open-shell molecules, but data curation and benchmarking need to be performed, which are currently underway. With these and other foreseeable improvements, the ML models are likely to facilitate the adoption of black-box automated reaction prediction methods to serve as a general tool for the chemical community.

## Data availability

The authors declare that the data supporting the findings of this study are available within the paper and its ESI files.† The code for this study is available through GitHub under the MIT License [https://github.com/zhaoqy1996/Delta2ML].

## Author contributions

Q. Z.: conceptualization, investigation, methodology, software, formal analysis, data curation, visualization, writing – original draft. D. M. A.: investigation, methodology, software, writing – original draft. O. I.: conceptualization, funding acquisition, resources, supervision. B. M. S: conceptualization, funding acquisition, resources, writing – review & editing, supervision.

## Conflicts of interest

The authors declare no conflict of interest.

## Supplementary Material

SC-014-D3SC02408C-s001
