# Supplementary material for: Δ2 machine learning for reaction property prediction
Source: Chem Sci. 2023 Jul 19;14(46):13392–401. doi: 10.1039/d3sc02408c (PMC10686042; doi:10.1039/d3sc02408c)
Supplement: SC-014-D3SC02408C-s001 [file SC-014-D3SC02408C-s001.pdf]

Electronic Supplementary Material (ESI) for Chemical Science.
This journal is © The Royal Society of Chemistry 2023

Δ^2 Machine Learning for Reaction Property Prediction

Supporting Information

Qiyuan Zhao[1], Dylan Anstine[2], Olexandr Isayev[2], and Brett M. Savoie[1*]

[1]*Davidson School of Chemical Engineering, Purdue University, West Lafayette, IN, 47906*

[2] *Department of Chemistry, Carnegie Mellon University, Pittsburgh, PA, 15213*

E-mail: bsavoie@purdue.edu

1 The Distribution of Geometric Deviations

Two measures are defined to quantify the geometric deviations between the low-level and high-level critical point geometries: mass-weighted root-mean-squared-displacement (RMSD) and maximum reactive bond length change (MBLC). Both RMSD and MBLC between the transition states optimized at GFN2-xTB and B3LYP-D3/TZVP levels of theory are mainly distributed among 0 and 1Å, which illustrates the rationality of setting the outlier threshold to 1Å(Fig. S1 a-b).

The uncertainty of the Δ^2 model can be estimated from the standard deviation of eight models that comprise the ensemble model. For 35137 of 36358 reactions (97%) in the test set, the prediction error is within three standard deviations (Fig. S1c), while the remaining 3% of reactions indicate a systematic inaccuracy. A residual number of unintended transition states being within the training and testing datasets cannot be ruled out, which may explain these errors. Some specific outliers are also discussed below.

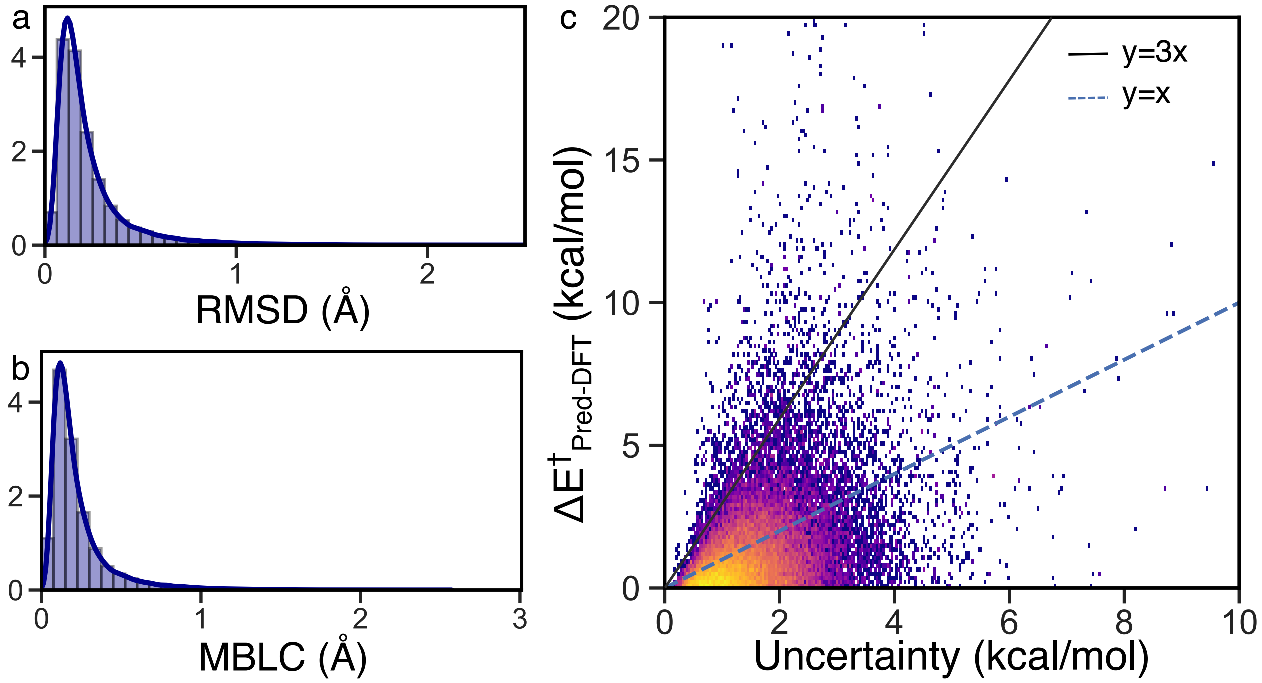

Figure S1: Statistics of the geometric deviations and prediction uncertainty. Distributions of root-mean-squared-displacement (RMSD, a) and maximum reactive bond length change (MBLC, b) of GFN2-xTB optimized and B3LYP-D3/TZVP optimized geometries. (c) Correlation between the prediction uncertainty estimated by the ensemble model and the actual deviation (i.e. reference DFT-level energy minus the average prediction of the ensemble model). Two lines (y=x and y=3x) are added to compare the actual deviation and the model uncertainty.

2 Prediction of Enthalpies of Reaction

In the main text, the activation energy is predicted by feeding the single-point energies of equilibrium structures (ESs) and transition states (TSs) into the Δ^2 model. Similarly, the enthalpies of reaction (ΔH_r) can be predicted by feeding the Δ^2 model enthalpies of equilibrium structures. Interestingly, the deviation of the ES prediction (MAE of 0.4 kcal/mol) is much smaller than the TS prediction (MAE of 1.2 kcal/mol, Fig. S2a) when predicting the single-point energy, indicating that the prediction of ΔH_r is an easier task compared to that of $\Delta E^{\dagger}$.

To train the ΔH_r model, the energy deviations change from $SPE_{DFT} - SPE_{xTB}$ into $H_{DFT} -$

SPE$_{xTB}$, where H$_{DFT}$ is computed as the single point energy plus the zero point energy correction and the room temperature thermal contributions to the molecular enthalpy estimated using a harmonic partition function calculated based on the normal mode frequencies.The model architecture remained unchanged. With a single ΔH_r model, the MAE reaches 0.58 kcal/mol, within the expected accuracy of DFT (Fig. S2b).

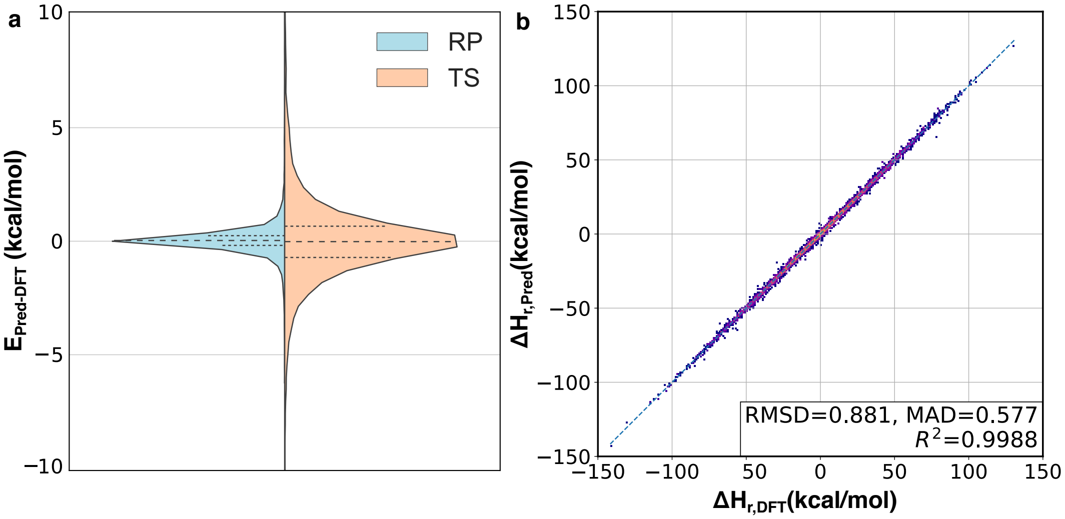

Figure S2: Prediction of enthalpies of reaction. (a) Error distribution of single point energy prediction on equilibrium structures (blue) and transition states (orange). (b) Bivariate kernel density estimations show the correlation between enthalpies of reaction computed using ground-truth DFT (x axes) and the Δ^2 model (y axes).

3 Analysis of Outliers

This section discusses reactions that are outliers in terms of the activation energy deviation between the ground-truth DFT and the Δ^2 model prediction. Reactions with deviations larger than 20 kcal/mol were regarded as outliers for this analysis.

In total 14 reactions (only counting the forward direction) have a $>$ 20 kcal/mol deviation in the predicted activation energy (Fig. S4), and these deviations are all derived from the TS single-point energy predictions. To analyze the source of this inaccuracy, the geometric differences

of GFN2-xTB optimized- and B3LYPD3-TZVP optimized-TSs were analyzed in detail (Fig. S4). Although both the RMSD and MBLC of these 14 pairs of geometries are smaller than 1Å, the comparison of specific TS geometries indicates that all but one TS (R7) have different chemistries at the two levels of theory. For example, in R1, the xTB-level TS includes an allene-like structure that leads to a linear alignment of the entire backbone, while the DFT-level TS is more flexible and stable. Similarly, in R2, the xTB- and DFT-level TSs contain three- and four-membered ring-like structures, respectively, which explains the overestimation of the TS energy using the xTB-optimized TS geometry as the model input. In addition, different reaction mechanisms of the two levels of the theory, such as different bond-breaking and bond-forming orders, also affect the energy prediction (e.g., R3, R4, R5, R8, R12). These outliers illustrate a limitation of the Δ^2 model, namely that it is difficult for the model to provide accurate predictions when the low level of theory is exhibits qualitatively different TS chemistry from the high level of theory.

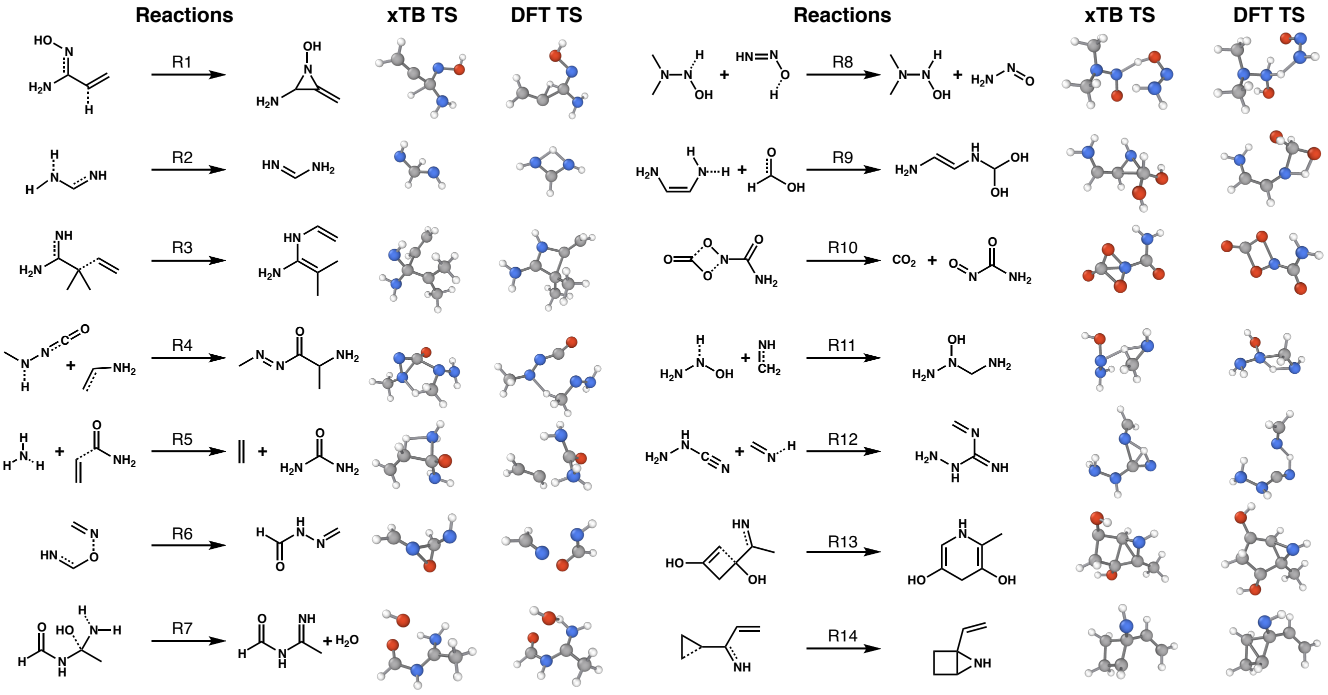

Figure S3: Geometric analysis of 14 outliers (deviation > 20 kcal/mol) with respect to the activation energy prediction of the Δ^2 model.

4 Timing Benchmarks

In order to assess the time cost involved in running the Δ^2 ML model, we conducted an ensemble model evaluation, composed of eight individual models, across four different systems (Table S1). These systems were chosen to represent a range of geometries (TSs), system sizes, and potential use cases. The time expense incurred was broken down into three components: data loading, model loading, and AIMNet2 execution. The initial two tasks were carried out on a single-CPU, while the final step was performed on a V100 GPU.

Table S1: Summary of Δ^2 model walltime and scaling behavior. All walltimes are reported without parallelization (i.e., single-core equivalent walltimes).

Test System	Number of TS	Data Loading (s)	Model Loading (s)	AIMNNET2 Executing (s)	Total Time (s)	Walltime per TS (ms)
Glucose	123	0.14	2.98	0.58	3.70	30.08
YARPv2.0	460	0.24	2.91	26.57	29.72	64.61
RGD1-10k	10,000	10.89	2.81	29.19	42.89	4.29
RGD1-100k	100,000	595.46*	2.89	45.01	643.36	64.34

The significant increase in AIMNet2 execution time when moving from the Glucose system to the YARPv2.0 system can be attributed to AIMNet2's method of bundling input geometries with the same number of atoms. As a result, the test transition states in the Glucose system, each containing 24 atoms, can be processed all at once. Additionally, the noticeable rise in data loading time in the RGD1-100k scenario indicates room for further improvement in the CPU-end workflow when dealing with large amounts of data. Conversely, model execution itself is stunningly fast in all cases.

5 Error Estimation of Δ Model

To estimate the minimum errors for a Δ model trained to predict energies at a given geometry rather than at different fixed-points, the deviations between the B3LYP-D3/TZVP//GFN2-xTB energies and the B3LYP-D3/TZVP//B3LYP-D3/TZVP energies were computed at 200 pairs of low-level/high-level TSs from RGD1. To ensure that these samples spanned the range of structural deviations available in the dataset, the transition states in RGD1 were sorted into five bins (evenly distributed from 0-1Å) based on RMSD and MBLC, the two metrics of geometric deviations. This produced two separate binnings of the RGD1 dataset, each with five bins. 20 TSs that were optimized at GFN2-xTB level of theory were randomly selected from each of the 10 bins for a B3LYP-D3/TZVP single-point energy evaluation, for a total of 200 structures. The associated B3LYP-D3/TZVP//B3LYP-D3/TZVP energies for each of these TSs were already available in the dataset. The deviations between the these single-point energies and the B3LYP-D3/TZVP//B3LYP-D3/TZVP energies were computed and presented in Figures 5c-d for comparison with the distribution of Δ^2 model errors over the testing dataset.

6 Other Figures

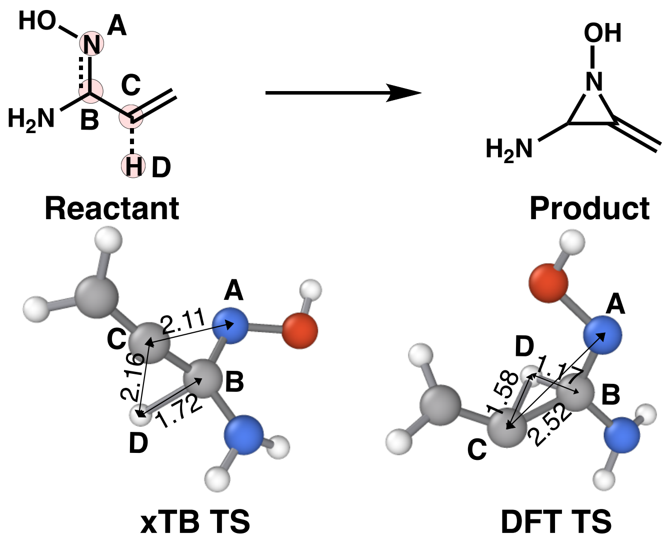

Figure S4: Illustration of the maximum reactive bond length change (MBLC) metric. The bond lengths of three reactive bonds, namely (A,C), (B,D) and (C,D), are computed for the GFN2-xTB optimized (bottom left) and DFT optimized (bottom right) TSs. In this case, the maximum bond length change is 0.58 Å, corresponding to the bond between atoms C and D.

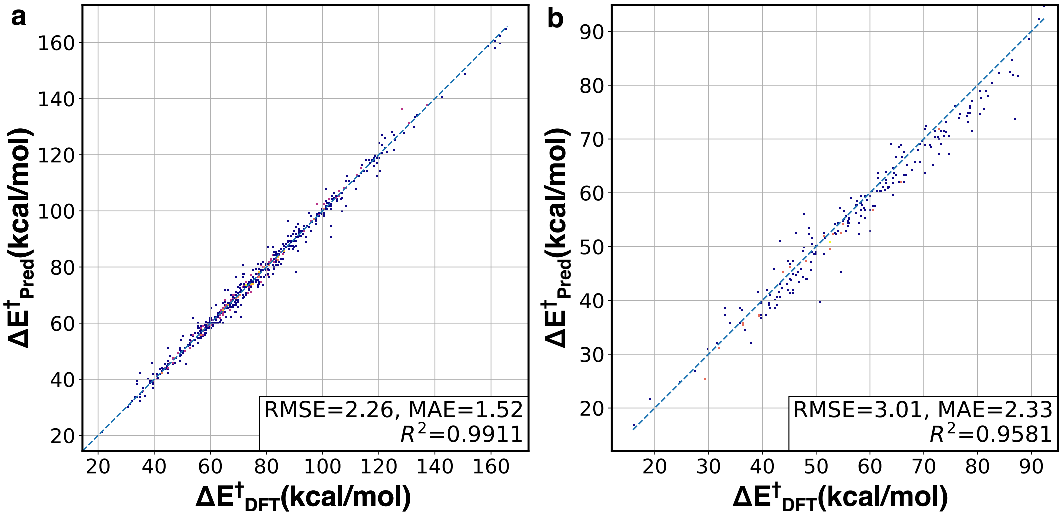

Figure S5: Performance of the Δ^2 model on external test sets. Bivariate kernel density estimations show correlation between activation energies computed at ground-truth DFT (x axes) and the Δ^2 model (y axes) when testing on (a) unimolecular decomposition networks and (b) glucose pyrolysis reactions. The units for mean absolute error (MAE) and root mean squared error (RMSE) are kcal/mol.

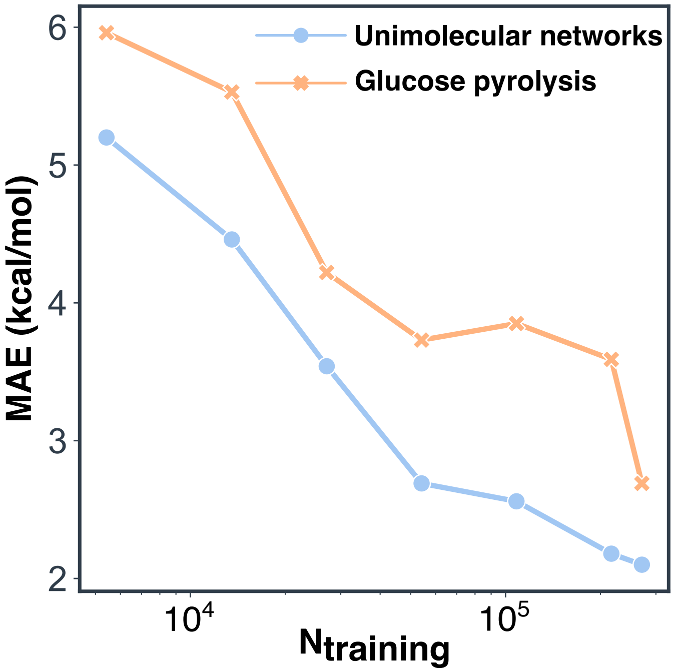

Figure S6: Performance analysis of the Δ^2 model on the external testing sets as a function of training data size.
